# Theory of Mind Impairments Highlighted With an Ecological Performance-Based Test Indicate Behavioral Executive Deficits in Traumatic Brain Injury

**DOI:** 10.3389/fneur.2019.01367

**Published:** 2020-01-22

**Authors:** Philippe Allain, Martin Hamon, Virginie Saoût, Christophe Verny, Mickaël Dinomais, Jeremy Besnard

**Affiliations:** ^1^Laboratoire de Psychologie des Pays de la Loire (EA 4638), University of Angers, Angers, France; ^2^Department of Neurology, Angers University Hospital, Angers, France; ^3^Arceau Anjou, Angers, France; ^4^Department of Physical and Rehabilitation Medicine, Angers University Hospital, Angers, France

**Keywords:** theory of mind, ecological assessment, behavioral dysexecutive disorders, traumatic brain injury, mentalizing abilities

## Abstract

**Background:** In view of the recent literature, the negative impact of traumatic brain injury (TBI) on social cognition remains a debated issue. On one hand, a considerable number of studies reported significant impairments in emotion recognition, empathy, moral reasoning, social problem solving, and mentalizing or theory of mind (ToM) abilities in patients with TBI. On the other hand, the ecological validity of social cognition tasks is still a matter of concern and debate for clinicians and researchers.

**Objectives:** The objectives of the present study were 2-fold: (1) to assess social cognition in TBI with an ecological performance-based test which focuses on ToM ability, and (2) to study the relationship between performances on this task and behavioral disorders. To this end, 47 patients with moderate to severe TBI in the chronic stage were assessed with a ToM task, the Movie for the Assessment of Social Cognition (MASC), a film displaying social interactions in natural settings and asking for an evaluation of the emotions, thoughts, and intentions of the characters. Behavioral disorders were assessed with the Behavioral Dysexecutive Syndrome Inventory (BDSI), a structured interview of an informant in assessing changes compared with previous behavior in 12 domains.

**Results:** Patients were significantly less accurate in mental state attribution than a demographically matched group of 38 healthy control subjects. Significant others of patients also reported more behavioral executive problems than controls' relatives on most of the domains of the BDSI. In addition, social cognition performance in the MASC was significantly correlated with behavioral dysexecutive problems rated by proxies on the BDSI.

**Conclusions:** This study is the first to find association between impairments in mentalizing abilities in the MASC and behavioral impairments in patients with TBI, confirming the added value of this ecological task and that the recognition of social signals is a key element for adequate behavioral functioning.

## Introduction

The notion of social cognition embraces several subdomains and refers to all the socio-emotional abilities and experiences regulating the relationships between individuals and allowing the explanation of individual human behaviors or behaviors in a group (1, 2). A core component of social cognition is Theory of Mind (ToM), namely, the ability to attribute mental states to ourselves and to others to explain and predict behavior (3). Social cognitive neuroscience [see, for example, (4, 5)] has defined two main subcomponents of ToM, including cognitive ToM (referring to beliefs, thoughts, and intentions) and affective ToM (referring to emotions and feelings).

ToM is a component of social cognition that is of concern for adults who suffered traumatic brain injury (TBI) as, over the past few decades, a proliferation of research has shown that adults with moderate to severe TBI exhibited significant deficits on ToM tasks [for review, see (6)]. These deficits have been observed for both components of ToM, early after injury (7–9) and in the chronic stage (10–12). These patients have difficulty understanding that someone else may have a wrong belief (13), identifying what may be embarrassing in a situation (14), detecting the intentions behind someone's behavior (7), and inferring what a person may think or feel (15).

For the purpose of our work, it is important to emphasize that most of the studies on ToM in TBI have been conducted using static scenario-based tasks, such as stories based on false belief or understanding a faux pas (8, 12, 16) or cartoon sequences based on intention predictions (7), in which one or more characters are presented with limited contextual information and participants are required to infer the mental states of the character(s) presented. Photographs of the eye region of the face have also been used (7). Although these tasks have been very helpful to understand the basic functioning of ToM, they often fail to really challenge healthy human's mentalizing capacity in a way like what happens on everyday basis in real life (17, 18). More specifically, these tasks lack ecological validity as they require participants to use their ToM abilities in static situations that are oversimplified, often unimodal (verbal or visual), relying on few indicators or cues, and finally very different from real-life situations. According to Achim et al. (17), a better way to assess ecologically ToM abilities is to use videos as stimuli as they present situations in a more naturalistic way (multimodal and dynamic) than verbal or visual static tasks.

Moderate to severe TBI also causes significant behavioral changes that may severely impact participation (19), return to work (20), quality of life (21), and caregiver burden (22). According to a recent review by Milders (23), the incidence of these changes is between 25 and 88% for persons with moderate to severe TBI, with higher prevalence rates associated with more severe TBI. These behavioral changes mainly include behavioral executive disorders (19) with, for example, hypoactivity, anticipation difficulty, euphoria, hyperactivity, environmental dependency, anosognosia, confabulation, and sexual conduct disorders [see, for example, (21, 24) for a description of the characteristics of the behavioral dysexecutive syndrome in cohorts of patients with severe or moderate to severe TBI].

Since these behavioral dysexecutive disorders often involve inadequate emotional behaviors or sociopathic behaviors [see, for example, (25)], deficits in social cognition have been put forward by several authors as a possible underlying mechanism [see, for example, (14, 23)]. In line with this proposition, Spikman et al. (26) found that deficits in basic emotion recognition after moderate to severe TBI, a core component of social cognition abilities, were related with behavioral changes reported by significant others in the Dysexecutive Questionnaire (27), a 20-item questionnaire measuring a broad spectrum of behavioral dysexecutive disorders. Recently, Milders (23) reviewed 10 studies [including the study by (26)] that examined correlations between recognition of emotions (in faces, in faces and tone of voice, or in dynamic face and body postures) and post-injury behavior in TBI (self-ratings or informant ratings concerning social communication, social integration, social outcome, and behavior). Six studies reported that better emotion recognition was significantly associated with fewer behavioral problems in these patients. In four studies, correlations were not significant, but in the expected direction.

Studies examining the relationships between ToM impairments and behavioral dysexecutive disorders in TBI are even rarer, the first being led by Milders et al. (14). In a seminal work, Milders et al. studied the relationships between ToM perception (assessed with a verbal ToM test, the faux pas test), and proxy ratings of difficulties in social/emotional behavior following TBI [assessed with the Neuropsychology Behavior and Affect Profile; (28)] and failed to find association between these variables. In a second study combining scores from the faux pas test and the cartoon test (a visual ToM test), Milders et al. (29) found no significant association between ToM impairments and proxy ratings of post-TBI social and emotional behavior changes using the same questionnaire. Similarly, in a third study, May et al. (30) found no significant association between proxy ratings of social behavior (with the Dysexecutive Questionnaire) following TBI and performance on four tasks of intention inferences (the faux pas test, the hinting test, the ToM cartoon test, the cartoon predictions test). In his recent review of literature, Milders (23) identified three other studies that correlated ratings of behavior following TBI with ToM abilities. In the study by Struchen et al. (31), an association between the ability to identify inappropriate behavior in video vignettes of social situations and self-ratings of social integration was reported in a group of 184 patients with TBI. In the study by Ubukata et al. (32), no significant correlation between ToM abilities (mind in the eyes test and faux pas test) and social outcome appeared. Finally, in the study by Byom and Turkstra (33), the better use of words that refer to thoughts, feelings, or desires was associated with a better quality of social communication (as rated by an independent observer) in moderate to severe TBI.

To sum up, few studies have investigated the putative links between ToM deficits and behavioral impairments in TBI. The available results are rather unconvincing or contradictory. As mentioned above, this could be related to the fact that ToM tests may lack ecological validity and may not be suitable to provide answers to questions with respect to daily life problems. These inconsistent results can also be explained by the diversity of questionnaires and the type of assessments (self-ratings vs. proxy ratings), the heterogeneity of samples (e.g., time since injury, number of participants), and the insufficient statistical power (23). Additionally, it should be noted that ToM ability is least routinely assessed than other processes of social cognition in clinical practice (34). Thus, additional studies are needed to support the evidence of an association between deficits in ToM and behavioral dysexecutive disorders. The present work is fully in line with this perspective. Our aim was to investigate whether ToM abilities, as measured with an ecological performance-based test, might be a predictor of behavioral dysexecutive deficits in patients who sustained a severe TBI, as measured by proxy ratings. We wanted to explore whether ToM impairments are related to behavioral dysexecutive disorders.

## Materials and Methods

### Ethics Statement

This study was approved by the local ethical research committees and the independent protection of individuals committee of University Ouest II, Angers, on 25 January 2013, and authorized by the National Health Authority on 29 January 2013. Written and oral information was given to the participants and their proxies. Written consent was obtained from all participants (patients, proxies, and legal representatives) when appropriate.

### Population

This work was part of AVEC-TC, a larger cohort study designed in our University Hospital to describe the treatment and management of individuals with moderate to severe TBI and the expertise of their proxies [see (24)]. In this cohort study, all patients with history of TBI using health or social services at the University Hospital of Angers, in local specialized rehabilitation or community-based facilities, or addressed to the investigators by the patient's family association, were screened for participation. Inclusion criteria in AVEC-TC were (1) existence of a moderate to severe TBI with an initial Glasgow Coma Scale (GCS) score of <13 and/or hospitalization for at least 48 h in intensive care; (2) participants were in the post-acute period (at least 3 months post-TBI); (3) participants were living in their own home or in a care facility; (4) with a proxy willing to participate in data collection and complete the behavioral evaluation. The proxy could be a relative, a friend, or a professional caregiver; (5) participants were aged between 18 and 65 years at the time of inclusion. Exclusion criteria included (1) non-traumatic acquired brain injuries, (2) mild TBI, and (3) speech or language impairments that would compromise the understanding of instruction and completion of the interviews and tasks. Additionally, in the present work, TBI patients with history of psychiatric problems were excluded.

All eligible participants were approached and asked to participate in AVEC-TC study. Data were collected via structured interviews. The participants and their proxies were convened by one of the investigators in a participating center. They were received together and then separately to complete questionnaires and tests. For professional caregivers, the questionnaires could be completed without the presence of an investigator and sent by mail.

The subgroup of patients with TBI who participated in our work included 47 individuals (29 males). Educational level ranged from 6 to 17 years of education [10.9 (2.8)]. Age at assessment ranged from 18 to 65 years [31.3 (10.5)], and age at injury ranged from 14 to 49 years [19.1 (4.9)]. The patients with TBI were, on average, 12.2 years post-injury at the time of the evaluation (SD = 10.21 years, range = 0.9–41 years). Mean coma duration was 18.2 (13.4) days (coma duration was not available in six cases), and mean post-traumatic amnesia duration was 42.6 (29.1) days (post-traumatic amnesia was not available for 11 cases). For 39 patients with TBI, GCS scores were available, ranging from 3 to 12, with a mean of 6.2 (2.2). At the time of the study, half of the patients with TBI (29/47) were in receipt of neuropsychological rehabilitation, but none of them was receiving or has received a rehabilitation program specifically focused on behavioral disorders. Forty patients with TBI were living at home, and the remaining patients lived in facilities specializing in the care of patients with brain damage.

Patients with TBI were compared to a group of 38 healthy control (HC) subjects (24 males) with a mean age of 31.2 years (range 19–57; SD 10.3) and a mean total year of education of 11.3 years (range 7–17; SD 2.3). All HC subjects were free of neurological and psychiatric illness and recruited from a database of volunteers.

### ToM Task

ToM abilities were measured with the Movie for the Assessment of Social Cognition [MASC; (35)], translated and validated into French in a partnership between the team of Dr. Patricia Garel (Sainte-Justine University Hospital Montreal, Québec) and the team of Dr. Isabelle Amado (Centre Hospitalier Sainte-Anne, Service Hospitalo-Universitaire, Paris, France) [see (36)].

The MASC includes a wide range of contexts/situations requiring ToM ability (37) and meets the criteria of ecological validity for mentalizing tasks proposed by Achim et al. (17). It consists of a short film of 15 min which describes four young protagonists (two females and two males) spending an evening together (one can see them cooking, eating, and playing games together). The MASC has the advantage of integrating visual and auditory input channels and to request online inferences based on visual cues such as facial expressions, gestures, and body language, like in real-life situations. Another advantage of the MASC is that it investigates inference of emotions (affective ToM), thoughts, and intentions (cognitive ToM) with a single task using comparable situations. The video is stopped at 45 moments during the story, and the subject must answer questions concerning the mental states of one of the characters (emotional epistemic, volitional), as well as to questions (*n* = 6) concerning non-mental details depicted in the video which are used to control for memory and general comprehension abilities. According to Dziobek et al. (35), 17 items assess the inference of emotions, seven items assess the inference of thoughts, and 18 items assess the inference of intentions. A typical question for the category inference of emotion is: “What is Ben feeling?”; for the subscale thoughts: “What is Anna thinking?”; and for the subscale intentions: “Why is Michaël saying this?” The subject must select his/her answers at the precise moment when the film is stopped among four possibilities: (1) correct answer (ToM), (2) “under-mentalizing” answer, (3) lack of mental state attribution answer (no ToM), and (4) “over-mentalizing” answer. Five main scores are derived from the MASC: (1) MASC sum of correct answers (maximum 45) as index of ToM performance (MASC ToM), three error scores; (2) “over-mentalizing” error score (Iper-ToM); (3) “under-mentalizing” error score (Ipo-ToM); (4) lack of ToM (No-ToM); and (5) score on control items (control score) as a measure of general comprehension ability (maximum six). Higher MASC ToM ccore and control score indicate better performance. Higher error scores (Iper-, Ipo-, and No-ToM) indicate lower performance. Administration of the tests takes between 30 and 45 min.

Many studies have shown that the MASC was a reliable and sensitive task for demonstrating subtle ToM impairments in individuals with social anxiety, body dysmorphic, or obsessive–compulsive disorders (38), or depressive subjects (39), in individuals with borderline traits (40), in adults with Asperger syndrome (35), and in patients with schizophrenia (36, 41). Studies in neurologic patients are rarer, with only two studies showing ToM impairments in multiple sclerosis (42, 43). Finally, Lecce et al. (37) have shown that older adults were less accurate in mental state attribution than young adults in the MASC, but not in more classical ToM tasks (strange stories, for example). The study herein is the first to use the MASC in a group of patients with TBI.

### Behavioral Executive Functioning

Behavioral dysexecutive deficits were assessed by proxies using the Behavioral Dysexecutive Syndrome Inventory (BDSI). This questionnaire is a part of the GREFEX battery (19). It proposes a structured interview that assesses changes compared to premorbid behavior in 12 different domains: (1) reduction of activities (hypoactivity with apathy-aboulia, avolition); (2) difficulties for anticipation, planning, and initiation of activities; (3) disinterest and indifference to his/her own concern and others; (4) euphoria, joviality, emotional lability; (5) irritability, aggressiveness; (6) hyperactivity, distractibility, impulsivity; (7) stereotyped and perseverative behavior; (8) environmental dependency; (9) anosognosia–anosodiaphoria; (10) confabulations; (11) social behavior disorders; and (12) disorders of sexual, eating, and urinary behavior. For each domain, the proxy is asked to state if the behavior differs from the participant's pre-injury behavior. If positive, the proxy is asked to rate the severity (from 1 to 3: mild, moderate, or major), frequency of occurrence (from 1 to 4: from occasional to daily), and the burden induced by the behavior (resounding score). To be considered as dysexecutive, behavioral disorders should not have other causes (cognitive, psychiatric, or sensorimotor disorders) and must significantly change the activities of daily life, social life, or work compared to the pre-injury state. The informant had to rate the frequency and the severity of behavioral changes, thus providing an index (frequency × severity) for each behavioral domain. According to Godefroy et al. (19), a domain should be considered as positive if the index is >2 (5% cutoff), and subjects with at least three positive domains could be considered to have a behavioral dysexecutive syndrome.

Proxies were close family members (spouses, mothers/fathers, brothers/sisters, children) for 40 (85%) participants with TBI, friends for 4 (8.5%), and professional caregivers for 3 (6.5%). Professional caregivers were paramedical professionals and educational or social workers. Proxies were close family members for 33 (87%) HC subjects and friends for 5 (13%). Proxy raters were required to have known the patients with TBI or HC subjects for at least 2 years and to have observed them in social situations. Please note that given the unequal sample sizes across the types of raters and the weakness of the samples of friends and professional raters, it was not possible to analyze whether ratings of behavioral problems differed according to rater type.

## Results

### Statistical Analyses

Tests for normality (Kolmogorov–Smirnov) indicated that the MASC subscores were normally distributed. Therefore, we used parametric tests (one-way and factorial ANOVAs) to evaluate differences between patients' performances with TBI and HC subjects for these scores. With significant factorial ANOVA results, *post-hoc* Scheffé tests were performed. As BDSI scores were non-normally distributed, we used non-parametric tests (Mann–Whitney *U* tests) to examine for behavioral differences between groups. Effect sizes (Cohen's *d*) were calculated for all comparisons between the groups. Regarding effect sizes for non-parametric statistics, as their estimates are known to be affected by departures from normality of variances, we followed the recommendations of Ivarsson et al. (44) [see also (45)] who suggested an effect size estimator for use in association with non-parametric statistics. To calculate the point–biserial correlation, these authors reported that the formula *r*pb = *z* / √*N* could be used, with *z* being the value obtained from Mann–Whitney *U*-test and *N* being the sample size. Next, the traditional Cohen's *d* value is calculated with the formula *d* = 2*r*/√(1-*r*^2^pb). According to Cohen's (46) suggestions (small: *d* = 0.20; medium: *d* = 0.50; large: *d* = 0.80), generally large effect sizes were found for significant differences between groups, whereas non-significant results were associated with small effect sizes. Spearman correlations were calculated to determine relationships between MASC scores and tBDSI-informant scores and between MASC scores and clinical data. Frequencies were compared with chi-square test. All statistical analyses were carried out using Statistica 9.0 (StatSoft. Inc. Tulsa, USA). The significance threshold was set at *p* < 0.01 rather than *p* < 0.05 to reduce the possibility of type I errors.

### Sociodemographic Characteristics

Chi-square and ANOVAs showed that patient and control groups were matched for sex (χ^2^ = 0.36, *p* = 0.54), age [*F*_(1, 83)_ = 0.01, *p* = 0.91] and educational level [*F*_(1, 83)_ = 0.35, *p* = 0.55].

### MASC Scores

Performances in the MASC are given in Table 1. There was no difference between groups for the MASC control score [*F*_(1, 83)_ = 1.01, *p* = 0.31]. Correct answers for the attribution of mental status (MASC ToM Score) were different between the two groups, with fewer correct answers in patients with TBI compared to HC subjects [*F*_(1, 83)_ = 171.36, *p* < 0.0001]. The effect size was very large according to Cohen (46).

**Table 1:** Performances of patients with TBI and HC subjects on the MASC (raw scores).

	**Patients with TBI (*n* = 47) mean (SD)**	**HC subjects (*n* = 38) mean (SD)**	p^*^	d^**^
MASC ToM Score correct (0–45)	23.1 (2.6)	31.8 (3.5)	<0.0001	1.40
MASC Control Score correct (0–6)	4.9 (1.2)	5.1 (0.8)	0.31	0.09
MASC error scores				
MASC Iper-ToM errors (0–45)	5.9 (2.5)	5.2 (2.1)	0.18	0.14
MASC Ipo-ToM errors (0–45)	13.1 (2.1)	5.4 (1.6)	<0.0001	2.06
MASC No-ToM errors (0–45)	2.8 (1.4)	2.6 (1.4)	0.34	0.09

*Mean and standard deviation (SD) of the various scores of the MASC test are indicated in the table. MASC ToM Score total number of correct answers, MASC Control Score number of correct answers for control questions, MASC Iper-ToM number of exceeding ToM errors, MASC Ipo-Tom number of less ToM errors, MASC No-ToM number of No-ToM errors*.

* *Overall analysis with ANOVA*,

** *effect sizes (Cohen's d)*.

Given that the MASC allows one to examine different aspects of mental state inference in a more ecological context, we were also interested in investigating whether TBI specifically impacted on the type of subcomponents of ToM in this context. To do that, we ran a two-way ANOVA on the percentage of correct answer on the MASC, with group (patients with TBI vs. HC subjects) as the between-subjects factor and type of mental state inference (emotion vs. thought vs. intention) as a the within-subjects factor. The main effect of group was highly significant [*F*_(1, 83)_ = 174.84; *p* < 0.0001]. Patients with TBI had lower percentages of correct inferences (mean 51.3%) than HC subjects (mean 70.6%) on the MASC across conditions. The main effects of type of mental state inference [*F*_(1, 83)_ = 0.04; *p* = 0.95] and the population × type of mental state inference interaction [*F*_(2, 166)_ = 0.54; *p* = 0.58] were not significant, showing that, independent of group, no differences on percentages of correct answers were found between inference of emotions (mean 65.4%), thoughts (mean 65.8%), and intentions (mean 64.7%) on the MASC. The absence of interaction reflected similar differences between proportions of correct answers for inferences of emotions, thoughts, and intentions in patients with TBI (thoughts vs. emotions, mean difference = 0.2%; thoughts vs. intentions, 1.9%; intentions vs. emotions, 2.1%) and HC subjects (thoughts vs. emotions, 1.2%; thoughts vs. intentions, 0.7%; intentions vs. emotions, 1.8%).

We were finally interested in investigating whether TBI specifically impacted on the type of errors made in attributing mental states. To this end, we performed a two-way ANOVA with group (patients with TBI vs. HC subjects) as a between-subjects factor and error type (Iper-ToM, Ipo-ToM, no-ToM) as a within-subject factor. Results showed a significant interaction between group and error type (*F*_(2, 166)_ = 50.63, *p* < 0.0001). We explored this interaction through pairwise comparisons. Results showed that patients with TBI reported a lower percentage of Iper-ToM errors (*p* < 0.0001; mean for patients with TBI, 27.1%; mean for HC subjects, 39.5%) and had higher percentages of Ipo-ToM (*p* < 0.0001; mean for patients with TBI, 59.8%; mean for HC subjects, 40.8%). No-ToM errors were less frequent (*p* < 0.0001) in patients with TBI (mean 13.0) than in HC subjects (mean 19.5). It is also important to note that within both groups, there were differences between all the error types, with the Ipo-ToM being the most frequent, the Iper-ToM being of medium frequency, and the No-ToM error being the least frequent. In the group of patients with TBI, the difference between percentages of Iper-ToM, Hypo-ToM, and No-ToM errors were significant (all *p* < 0.0001). In HC subjects, the percentages of Iper-ToM and Ipo-ToM errors did not significantly differ (*p* = 0.91), and both were significantly higher than the percentage of no-ToM errors (all *p* ≤ 0.0001).

### Behavioral Dysexecutive Syndrome Inventory

On the BDSI, higher scores equate to more behavioral problems. The proxies/professional indexes (frequency × severity) appeared higher in all behavioral domains, with significant differences between patients with TBI and HC subjects for 10 indexes (see Table 2): reduction of activities (*U* = 291.5, *z* = −5.31, *p* < 0.0001), anticipation–planning–initiation disorders (*U* = 374.5, *z* = −4.58, *p* < 0.0001), disinterest and indifference (*U* = 439.5, *z* = −4.00, *p* < 0.0001), euphoria–joviality–emotional lability (*U* = 617, *z* = −2.43, *p* = 0.01), irritability–aggressiveness (*U* = 399.5, *z* = −4.36, *p* < 0.0001), hyperactivity–distractibility–impulsivity (*U* = 535, *z* = −3.16, *p* = 0.0001), stereotyped and perseverative behavior (*U* = 468.5, *z* = −3.75, *p* = 0.0001), anosognosia–anosodiaphoria (*U* = 401.5, *z* = −4.34, *p* < 0.0001), social behavior disorders (*U* = 437, *z* = −4.03, *p* < 0.0001), and disorders of sexual–eating–urinary behavior (*U* = 589, *z* = −2.68, *p* = 0.007). The differences did not reach significance for environmental dependency (*U* = 829, *z* = −0.56, *p* = 0.57) and confabulations (*U* = 839, *z* = −0.47, *p* < 0.63). The effect sizes for the significant differences ranged from −0.54 to −1.41, which can be classified as moderate to very large according to Cohen (46).

**Table 2:** Proxies'/professionals' indexes (frequency × severity) for patients with TBI and HC subjects on the behavioral domains of the BDSI (mean and standard deviation).

	**Patients with TBI (*n* = 47) mean (SD)**	**HC subjects (*n* = 38) mean (SD)**	p^*^	d^**^
Reduction of activities	5.5 (4.4)	0.3 (0.6)	<0.0001	−1.41
Anticipation–planning–initiation disorders	4.2 (3.7)	0.9 (1.8)	<0.0001	−1.14
Disinterest and indifference	3.1 (3.3)	(0.5) (1.4)	<0.0001	−0.96
Euphoria–joviality–emotional lability	1.7 (2.4)	0.3 (0.7)	0.01	−0.54
Irritability–aggressiveness	2.9 (3.0)	0.3 (0.8)	<0.0001	−1.07
Hyperactivity–distractibility–impulsivity	2.5 (3.5)	0.3 (0.9)	0.0004	−0.73
Stereotyped and perseverative behavior	3.1 (4.1)	0.2 (0.7)	<0.0001	−0.89
Environmental dependency	0.2 (0.6)	0.1 (0.4)	0.57	−0.12
Anosognosia–anosodiaphoria	4.7 (4.7)	0.3 (1.0)	<0.0001	−1.06
Confabulations	0.2 (0.4)	0.1 (0.4)	0.63	−0.10
Social behavior disorders	3.7 (4.3)	0 (0)	<0.0001	−0.97
Disorders of sexual–eating–urinary behavior	1.5 (2.5)	0 (0)	0.007	−0.60

* *Mann –Whitney U-tests*,

** *effect sizes (Cohen's d)*.

Using a 5% cutoff (19), behavioral indexes were impaired in 10–62% of patients with TBI. Frequency of impairment ≥50% was observed for reduction of activities (62%), anosognosia–anosodiaphoria (56%), and anticipation–planning–initiation disorders (54%). Frequency of impairment ≤ 50% was observed for disinterest and indifference (49%), social behavior disorders (49%), irritability–aggressiveness (47%), stereotyped and perseverative behavior (43%), disorders of sexual–eating–urinary behavior (35%), euphoria–joviality–emotional lability (32%), hyperactivity–distractibility–impulsivity (32%), environmental dependency (10%), and confabulations (10%). Thirty-seven patients with TBI (79%) had a behavioral dysexecutive syndrome.

### Correlations

For patients, the correlations (Spearman correlation coefficients) between medical data and the performance on the MASC (MASC ToM Score) were non-significant: duration of coma (rho = 0.24, *p* = 0.15), post-traumatic amnesia (rho = 0.31, *p* = 0.07), and mean time since injury (rho = −0.007, *p* = 0.96).

We investigated the relationships between ToM impairments and behavioral dysexecutive disorders in patients. Since we did not find any effect of the specific subdomains of ToM (emotions, thoughts intentions) on the performance of patients, and to limit the number of correlations, we were only interested in the relationships between the total number of correct answers on the MASC and BDSI indexes. As expected, significant correlations were observed between MASC and BDSI scores. More specifically, in patients with TBI, there were significant correlations between the MASC ToM Score and proxies'/professionals' indexes for reduction of activities (rho = −0.46; *p* = 0.0009), disinterest and indifference (rho = −0.47; *p* = 0.0007), hyperactivity–distractibility–impulsivity (rho = −0.45; *p* = 0.001), irritability–aggressiveness (rho = −0.34; *p* = 0.01), and social behavior disorders (rho = −0.44; *p* = 0.0001). All correlations were negative, indicating that poorer performance on the MASC corresponded with more problems on the BDSI. Nevertheless, no significant correlation emerged between MASC ToM Score and BDSI proxies'/professionals' indexes for anticipation–planning–initiation disorders (rho = −0.25; *p* = 0.08), euphoria–joviality–emotional lability (rho = 0.006; *p* = 0.96), stereotyped and perseverative behavior (rho = −0.08; *p* = 0.55), environmental dependency (rho = 0.07; *p* = 0.61), anosognosia–anosodiaphoria (rho = −0.08; *p* = 0.55), confabulations (rho = −0.01; *p* = 0.92), and disorders of sexual–eating–urinary behavior (rho = 0.12; *p* = 0.39).

In HC subjects, correlations between the MASC ToM Score and proxies' indexes were significant for social behavior disorders (rho = 0.50; *p* = 0.002), euphoria–joviality–emotional lability (rho = 0.42; *p* = 0.009), stereotyped and perseverative behavior (rho = 0.45; *p* = 0.005), anosognosia–anosodiaphoria (rho = 43; *p* = 0.008), and disorders of sexual–eating–urinary behavior (rho = 0.50; *p* = 0.002). Correlations were non-significant for reduction of activities (rho = 0.01; *p* = 0.91), disinterest and indifference (rho = 0.19; *p* = 0.23), hyperactivity–distractibility–impulsivity (rho = 0.29; *p* = 0.07), irritability–aggressiveness (rho = 0.24; *p* = 0.13), anticipation–planning–initiation disorders (rho = 0.06; *p* = 0.68), environmental dependency (rho = 37; *p* = 0.02), and confabulations (rho = 26; *p* = 0.10).

## Discussion

To the best of our knowledge, this is the first study that found ToM impairments after moderate to severe TBI using the MASC, a single dynamic task that featured a combination of verbal and visual content in a social context, to conduct an ecologically valid assessment of daily life social interactions. We confirm that the French version of the MASC is a sensitive test in capturing deficits in attribution of mental states. In line with previous findings, we observed that patients with moderate to severe TBI were significantly impaired for both affective (emotions inferences) and cognitive (inference of thoughts and intentions) components of ToM abilities when compared to a matched group of HC subjects. In both groups, there was no significant difference between the proportions of correct answers for cognitive and affective items. In line with past literature, this could suggest an equivalent decline in ToM performances in TBI (7, 10–12, 26). The fact that patients show difficulties in the attribution of the right mental states to others in such a real-life social scenario suggests that their decay in ToM performance is not simply due to the limited ecological validity of the tasks usually used. This decay can be considered as a genuine deficit that reflects a real decline in ToM abilities.

In this work, the use of the MASC to investigate ToM also allowed us to examine the type of errors that patients with TBI make when they wrongly attribute mental states to others. Our results revealed that they produce more Ipo-ToM and No-ToM errors than HC subjects, suggesting that moderate to severe TBI reduces mental state attribution. Under-mentalizing behaviors have also been observed in patients with multiple sclerosis (42, 43), suggesting that brain lesions diminish the abilities to attribute mental states to others. This profile of errors (lack of ToM inferences) has also been observed among patients with psychiatric disorders, such as patients with schizophrenic and autism spectrum disorders [see, for example, (36, 41)]. Our findings that moderate to severe TBI is characterized by insufficient mental state reasoning for emotions, thoughts, and intentions in ecological settings add to the existing literature on the presence of social cognition impairments in TBI and expand the profile of TBI. These results can be helpful to orient treatments of ToM impairments. These rehabilitation programs must focus on mental states attribution rather than on an inclination to “over-mentalize,” which seems to be not the principal ToM deficits of patients with TBI, contrary to some psychotic spectrum conditions such as schizophrenia [e.g., (47)].

In line with previous findings, and according to the BDSI, more than three quarters of the sample (79%) had dysexecutive behavioral disorders, which is consistent with past findings. In their study, Azouvi et al. (21) found a prevalence of 81.5% of behavioral dysexecutive syndrome in a population of individuals with severe TBI, with a very similar distribution in the sub-domains of BDSI. Indeed we also observed that reduction of activities, anticipation–planning–initiation disorders, and anosognosia–anosodiaphoria were the most frequent behavioral changes after TBI (frequency of impairment ≥50%). In our study, disinterest and indifference, social behavior disorders, and irritability–aggressiveness were also very frequent behavioral changes reported by professionals and closest relatives as compared to pre-injury. Estimated rates for disinterest and indifference [49% in this study and 46.3% in the study by Azouvi et al. (21)] or irritability–aggressiveness (47 vs. 42.6%) were close to those found by Azouvi et al. (21). For social behavior disorders, the estimated rate was higher in our study (49 vs. 24.1%).

Our study is very clearly in favor of the idea that deficits in social cognition may contribute, at least in part, to executive behavioral disorders. In fact, we observed important relationships between the MASC ToM score and various indexes of the BDSI (reduction of activities, disinterest and indifference, hyperactivity–distractibility–impulsivity, irritability–aggressiveness, and social behavior disorders). Our results are in line with what was expected in this work and are clearly in favor of the idea that the MASC is an ecologically valid test of social cognition impairments. Our results are also in accordance with the models of social cognition that propose that social cognition abilities are important for social functioning and that impairments in social cognition abilities may result in difficulties with social behavior (48–50).

Reduction of activities (hypoactivity with apathy–aboulia, avolition) was associated with ToM performance. This finding suggests that hypoactivity also reduces mental state attribution. In the present study, the fact that patients presented under-mentalizing behaviors (producing more Hypo- and No-ToM errors than HC subjects and more Hypo- and No-ToM errors than Hyper-ToM errors) is consistent with this proposition. In addition, neuropsychological investigations have already documented an association between apathetic manifestations and low performance on tests assessing ToM abilities in some neurological and psychiatric disorders (51–56). Disinterest and indifference are associated with impairment in ToM. This finding suggests that these manifestations prevent patients from taking into consideration others' points of view and is consistent with the view that the ability to move from an egocentric perspective to an exocentric perspective is crucial in ToM (57). The fact that hyperactivity–distractibility–impulsivity and irritability–aggressiveness were associated with ToM performance could be interpreted in the same way. These behavioral manifestations surely have to do with an inability to break away from environmental stimuli (hyperactivity–distractibility–impulsivity) or from personal preoccupations (irritability–aggressiveness) or, in other words, with an inability to disengage from environmental stimuli and one's own perspective and consider others' points of view. Finally, attribution of mental states was found to be strongly associated with social behavior disorders. This finding suggests that impaired inference of emotions, thoughts, and intentions contributes to the occurrence of social behavior disorders. Under-mentalizing could lead patients to misunderstand the internal mental states of their interlocutors and therefore not to adapt and/or adjust their own behaviors toward these interlocutors.

In the control group, correlations between MASC ToM score and behavioral ratings do not went in the same direction, confirming that social cognition indexes sometimes behave differently in the presence/absence of TBI. Significant coefficients were positive, which makes them difficult to interpret: the better the HC subjects were at the MASC, the more their relatives considered they had behavioral problems. In addition, with respect to significant correlations, no proxies of control subjects reported social behavioral disorders (0//38) and disorders of sexual–eating–urinary behaviors (0/38). Concerning the three other BDSI indexes (joviality–emotional lability, stereotyped and perseverative behavior, anosognosia–anosodiaphoria), only two to five proxies scored them differently from 0. Regarding these elements, the validity of these correlations and their meaningfulness seem questionable to us.

Some limitations of our study must be considered. Firstly, there may have been some selection bias due to our inclusion criteria. Indeed we enrolled patients living in the care or with a proxy. Participants who were not in contact with health institutions or who were living alone were not included. This may have led to an overestimation or an underestimation of behavioral dysexecutive impairments. However, this type of bias is inherent to the assessment of individuals with brain lesions. Secondly, we could not guarantee that all participants were free from personality problems that might have influenced their ToM abilities, such as a lack of empathy, an inability to understand other people's emotions, or alexithymia. However, none of the patients that we included had a history of psychiatric problems according to medical records and anamnesis with patients and proxies. Thirdly, in the same vein, 11 patients incurred their injuries before age 18 (between 14 and 16 years), at a developmental milestone that could have impacted upon the development of social to us emotional skills [see, for example, (58)]. So we could not guarantee that all patients with TBI were free from developmental problems that might influence their ToM performance. Fourthly, we did not propose a neuropsychological assessment to patients with TBI, in particular of non-social cognitive functions frequently impaired in this population: speed of information processing, attention, executive functions, and working memory. Impairments of these functions in the group of patients with TBI could potentially contribute to their poorer performance on the MASC since it is a multimodal and dynamic ToM task presumed to strongly appeal to cognitive skills. However, this was not possible in the AVEC-TC study. In addition, we did not consider this possibility very likely since results of several studies document a possible dissociation between cognitive impairment after severe acquired brain injury and social cognition deficits [see, for example, (7, 59–62)]. In this regard, some authors have suggested that ToM and other cognitive domains should be considered as independent systems (7, 8, 63). In line with this argument, Laillier et al. (64) found that cognitive measures partially mediated the age effect on cognitive and affective ToM performances in healthy subjects using the MASC. In addition, dissociations between cognitive and behavioral assessments have been found in TBI with patients performing within the normal range on the cognitive battery while demonstrating significant behavioral changes (21). Such findings indicate that cognitive and behavioral dysexecutive syndromes may be dissociated (19) and support the hypothesis that behavioral disorders cannot always be explained by cognitive disorders. Another limitation concerns the fact that ToM was assessed with a single video-based task, namely, the MASC. Other tasks of this type are available [see, for example, the Video Social Inference Task; (65, 66)]. We selected this test because of its ecological validity, its ability to examine different subdomains of ToM as well as its high sensitivity. However, we must keep in mind that the MASC remains an offline paradigm of social cognition that focuses on ToM from an observer's rather than from an interactor's point of view [for the distinction between online and offline tasks of social cognition, see (67)]. In future works, adding online paradigms of ToM, with direct person-to-person interactions [for an example of online ToM tasks, see (68)], would surely enhance further ecological validity and help us to better understand the nature of the links between ToM deficits and social behavioral disorder in TBI. A final point concerns the use of an informant assessment of behavioral disorders. Indeed hetero-evaluations may lead to overestimation or underestimation of behavioral problems, depending, for example, on the burden induced by the behavior (69). However, behavioral disorders are often more precisely described by proxies than by patients because of anosognosia. In the same logic, it may well be that assessment of behavioral problems could naturally differ depending on the type of rater employed (i.e., relative vs. professional), suggesting that, in future studies on the relations between ToM deficits and behavioral dysexecutive disorders, it would be certainly important to obtain behavioral assessments provided from different types of raters. If the correlations we found in this work were confirmed through behavioral assessments made by different types of raters, they would have more weight. The opposite would allow us to bring nuances to our conclusions.

In conclusion, the main findings of the present study revealed that patients with moderate to severe TBI were less accurate to attribute emotions, thoughts, or intentions to characters than HC subjects in an ecologically valid ToM task. They also made more Hypo-ToM and No-ToM errors than HC subjects. ToM deficits were linked to behavioral executive dysfunctions. Our data are consistent with the view that ToM impairments might be a predictor of behavioral dysexecutive deficits in patients who sustained a moderate to severe TBI However, further investigations with larger samples of persons with TBI will be necessary to determine if the relationship between affective and cognitive ToM impairments and behavioral changes in a patient is causal or not.

## Data Availability Statement

The datasets generated for this study are available on request to the corresponding author.

## Ethics Statement

The studies involving human participants were reviewed and approved by the local ethical research committees and the independent protection of individuals committee of University Ouest II, Angers, on 25 January 2013, and authorized by the National Health Authority on 29 January 2013. The patients/participants provided their written informed consent to participate in this study.

## Author Contributions

PA, VS, and MD conceived and planned the experiment. VS and MD recruited participants. MH was involved in acquisition of data. PA, MH, and JB processed the experimental data and performed the analysis. PA wrote the manuscript with assistance from JB, MD, VS, and CV. PA supervised the project. All authors commented on the manuscript.

### Conflict of Interest

The authors declare that the research was conducted in the absence of any commercial or financial relationships that could be construed as a potential conflict of interest.
